# Use of Household Apparent Food Intake Data to Estimate Micronutrient Inadequacy in Comparison to the 24-h Recall Data Among Women of Reproductive Age in Kasungu District, Malawi

**DOI:** 10.3390/nu17152485

**Published:** 2025-07-30

**Authors:** Alexander A. Kalimbira, Zione Kalumikiza-Chikumbu, Gareth Osman, Bridget Mkama, Edward J. M. Joy, Elaine L. Ferguson, Lucia Segovia de la Revilla, Louise E. Ander, Sarah Pedersen, Omar Dary, Jennifer Yourkavitch, Monica Woldt

**Affiliations:** 1 Department of Human Nutrition and Health, Bunda College, Lilongwe University of Agriculture and Natural Resources, Lilongwe P.O. Box 219, Malawi; zkalumikiza@luanar.ac.mw (Z.K.-C.); gosman@luanar.ac.mw (G.O.); briemkama@gmail.com (B.M.); 2 Faculty of Epidemiology and Population Health, London School of Hygiene & Tropical Medicine, Keppel Street, London WC1E 7HT, UK; edward.joy@lshtm.ac.uk (E.J.M.J.); elaine.ferguson@lshtm.ac.uk (E.L.F.);; 3 School of Biosciences, University of Nottingham, Sutton Bonington Campus, Loughborough LE12 5RD, UK; 4 USAID, Bureau for Resilience, Environment, and Food Security, 1300 Pennsylvania Avenue, NW, Washington, DC 20523, USA; 5 USAID, Bureau for Global Health, 1300 Pennsylvania Avenue, NW, Washington, DC 20523, USA; 6 USAID Advancing Nutrition, 4th Floor, 2733 Crystal Drive, Arlington, VA 22202, USAmonica.woldt0803@gmail.com (M.W.); 7 Results for Development, 1111 19th Street, NW, Suite 700, Washington, DC 20036, USA; 8 Helen Keller International, 4th Floor, OAS Building, 1889 F Street, NW, Washington, DC 20006, USA

**Keywords:** 24-h dietary recall, fortification, household consumption and expenditure survey, micronutrients, Malawi

## Abstract

Objective: The aim of this study was to compare micronutrient intake and inadequacy estimates using household consumption and expenditure survey (HCES) and quantitative 24-h recall (24HR) data among women of reproductive age (WRA) in Kasungu district, Malawi. Methods: We conducted a secondary data analysis utilizing HCES dietary data from a subsample of households in rural areas of Kasungu district, which were sourced from the 2019/20 Malawi Fifth Integrated Household Survey (*n* = 183); and 24HR data were obtained from WRA in a community-based Addressing Hidden Hunger with Agronomy (AHHA) trial in the same district (*n* = 177). Micronutrient intakes and inadequacy were estimated under two alternative scenarios of large-scale food fortification (LSFF). We standardized apparent nutrient intakes from the HCES data using the adult female equivalent metric. Results: Estimated prevalence of micronutrient inadequacy fell within 20 percentage points between HCES and 24HR for iron (Fe), zinc (Zn), vitamins B2 and B9 under both no fortification and fortification scenarios. There were some discrepancies for the remaining B vitamins, being consistently large for vitamin B3. Conclusions: In the absence of 24HR data, HCES data can be used to make inferences about some micronutrient intakes and inadequacies among rural WRA in Malawi and to inform decisions regarding LSFF, including vehicle selection and coverage. However, additional efforts are needed to improve HCES for dietary nutrient surveillance given existing limitations.

## 1. Introduction

Dietary assessment is a method of assessing the nutritional status of individuals that focuses on evaluating food and nutrient intake and dietary patterns of individuals, households, and populations [1]. The Food and Agriculture Organization of the United Nations (FAO) categorizes dietary assessment into direct (e.g., 24-h recall [24HR]) and indirect methods (e.g., household consumption and expenditure survey [HCES]) [2]. An inventory of dietary assessment methods used in Africa revealed that 24HRs and food frequency questionnaires are most frequently used, although with low coverage of some population strata. Few studies have been conducted to compare results from intake surveys against biomarker indicators of nutritional status [3].

The 24HR is a structured interview that generates quantitative data on foods and beverages consumed by an individual during the previous 24 h [3]. Researchers can use data from a 24HR survey alongside information from a food composition table to estimate outcomes such as food and nutrient intakes, dietary patterns, and dietary adequacy [4]. Compared to indirect methods used to generate food consumption data, the 24HR is generally considered to have a high degree of accuracy and comprehensiveness [5]. However, 24HR surveys are complex and costly, and they are rarely conducted at a national scale or designed to capture seasonal variation in diet. Because the method is often based on recall over a small number of days (often a single day), it may poorly represent the consumption of episodically consumed foods. These constraints limit the use and applicability of 24HR survey data, which, while it can be repeated for several days, is often collected for a single day because it is more resource-expensive [5].

In the absence of nationally representative 24HR data, HCES may provide a valuable source of food consumption data. HCES, including those conducted as part of the Living Standards Measurement Study (LSMS) of the World Bank, recruit large samples of households designed to be representative at national and subnational levels [6,7]. The primary purpose of HCES is to generate information on household welfare and poverty, and they are routinely conducted every three to five years. HCES typically include a module that collects information on food consumption, food acquisition, and/or food expenditure. The Malawi HCES, commonly referred to as the Integrated Household Survey (IHS), asks a single member of the household with responsibility for food preparation to recall food items (using a fixed food item list) consumed by all household members (in aggregate) over a 7-day recall period. Since 1997, Malawi has implemented five HCESs, but no nationally representative food consumption surveys using a 24HR have been done [8]. While the HCES is not designed to measure food and nutrient intakes at the individual level, household food consumption data can be ‘individualised’ to provide a proxy measure of food and nutrient intakes [9]. In addition, researchers can use HCES data to estimate reach, potential contributions, and equity dimensions of large-scale food fortification (LSFF) programs for improving dietary micronutrient supplies and reducing risks of inadequate micronutrient intake by household characteristics [10].

The aim of the study was to compare micronutrient intakes and inadequacy estimates for women of reproductive age (WRA) using data derived from HCES and 24HR datasets to determine agreement between the two methods when assessing nutrient intakes and adequacy. We also explored whether the results from the two data sources lead to similar conclusions to inform LSFF programming. These analyses were undertaken to determine whether HCES data can be used to make dietary inferences when 24HR data are not available. The current study utilised 24HR dietary recall data generated among adult women participating in a recent community-based trial in rural Malawi. Thus, for comparability, the HCES data were ‘individualised’ using the adult female equivalent approach, to estimate apparent food and nutrient intakes among adult women [11].

## 2. Methods

### 2.1. Estimating Apparent Food Consumption Using HCES Data

We derived household food consumption data from the Fifth Integrated Household Survey (IHS5), a comprehensive and nationally representative survey implemented by the National Statistics Office (NSO) of Malawi. We obtained the IHS5 data from the World Bank’s open-data repository [12]. The NSO implemented IHS5 nationwide between April 2019 and April 2020. A stratified two-stage sampling design was used based on the cartography from the 2018 Malawi Population and Housing Census. The Malawi NSO selected 12,288 households from 768 enumeration areas (EAs). Due to the COVID-19 pandemic, 51 EAs (854 households) were not visited by the end of the 12-month data collection exercise. The aggregation resulted in a final sample size of 11,434 households, which was statistically representative at the national, district, and urban/rural residence levels. In the current study, we selected only households from the Kasungu district (n = 384) for comparison with the 24HR data. This was further refined based on the month of data collection (see below).

Apparent food intake data were collected as part of an IHS5 household questionnaire module. We utilized the file ‘household_module_g1 (hh_mod_g1)’, which recorded household food intake based on a predefined list of 131 items and others to specify based on food category. Specifically, one member of the household responsible for food acquisition and preparation was asked to recall, “Over the past 7-days, did you or others in your household consume any (item)? How much in total did your household consume in the past 7-days?”. Notably, the method does not capture foods consumed away from home. Food consumption quantities were divided by seven to generate estimates of daily apparent intake for the household.

The HCES food consumption data were ‘individualized’ to generate an estimate of apparent food intake based on the adult female equivalent (AFE) approach, calculated using ‘household_module_B (hh_mod_B)*’* for the household roster (individual-level age-gender data). Consistent with previous studies, we use the qualifier ‘apparent’ to emphasize that intakes were not directly measured; rather, they were approximated from household-level dietary information. The AFE approach divides household-level food consumption among members of the household, assuming each individual’s intake is proportional to their energy requirements [13], standardized to a nonpregnant, nonlactating 18- to 29-year-old woman as the reference household member [10]. To calculate the AFE metric, we estimated individual energy requirements using age- and sex-specific body weights and physical activity levels (PALs) informed by nationally representative and district-relevant data sources.

For women aged 18–29.9 years, an average body weight of 55.4 kg was used, based on the 2015/16 Malawi Demographic and Health Survey [14]. For all other adult age groups, average body weights were derived from a large cross-sectional non-communicable disease (NCD) study conducted in both rural (Karonga district) and urban (Lilongwe city) areas in Malawi between 2013 and 2016 [15]. Given that our study setting was rural Kasungu district, we used data from Karonga district (rural sample, n = 13,878). The following average body weights were applied: women—57.9 kg (30–59.9 years) and 55.2 kg (≥60 years), and men—58.2 kg (18–29.9 years), 59.7 kg (30–59.9 years), and 57.2 kg (≥60 years). For children under 18 years, age- and sex-specific body weights and PALs were obtained from FAO/WHO guidelines [16].

PAL values for adults were stratified by sex and age, informed by an accelerometer-based study conducted in rural Malawi [17], which reported high levels of physical activity consistent with a moderately to very active lifestyle (likely falling between 1.75 and 1.99 PAL). Therefore, the following PAL values were assigned: women—1.85 (18–59.9 years) and 1.75 (60+ years), while men—1.90 (18–59.9 years) and 1.75 (60+ years). To introduce realistic variability and avoid bias from fixed assumptions, we implemented a simulation-based imputation approach. For each age-sex group: body weight was simulated with a standard deviation (SD) of 5 kg, while PAL was simulated with an SD of 0.1. This process was repeated 100 times, generating 100 imputed datasets. In each simulation, a new set of weight and PAL values was generated for each age-sex group. The final values used in energy requirement calculations were the mean of the 100 simulated values for each group.

Basal Metabolic Rate (BMR) was estimated using FAO’s Table 5.2 equations based on body weight, and total energy requirements were calculated by multiplying BMR by the assigned PAL [16]. For pregnant and lactating women, an additional +300 kcal/day and +500 kcal/day, respectively, were added to the base energy requirement [18]. Because the Malawi HCES did not record breastfeeding status of young children, we assumed all children <24 months of age in the household roster were breastfeeding and the child’s mother was lactating to make inferences on energy requirements, as was performed previously by Tang et al. [10]. We selected a WRA as the reference member of a household to enable comparison with the 24HR intake data, which was collected among WRAs.

### 2.2. Estimating Food Consumption from 24HR Dietary Data

The 24HR data were collected as part of the baseline survey for the Addressing Hidden Hunger with Agronomy (AHHA) trial, a double-blind, randomized, controlled trial designed to test the effectiveness of biofortified maize for addressing selenium deficiency, implemented in Traditional Authority Wimbe, a rural community in Kasungu district, Malawi. The trial protocol and its outcomes have been reported elsewhere [19,20,21]. Briefly, households with at least a WRA aged 20–45 years who lived with a child 5–10 years of age meeting eligibility criteria were listed, and a random sample was selected for recruitment. In total, 180 households agreed to participate in the study, with one WRA and one child recruited in each household following provision of written informed consent. The baseline survey, including dietary data collection among WRA, was conducted in June–July 2019.

Using a four-pass interactive 24HR, the researchers collected dietary data from participating WRA to estimate the percentage of WRA at risk of selenium deficiency due to inadequate dietary intakes. In the first pass, enumerators asked participants to recall all foods and beverages consumed in the 24 h preceding the survey. Detailed descriptions of foods and beverages, including the time and place of consumption, were collected in the second pass. Estimates of the quantity of each food and beverage consumed were determined in the third pass using an interviewer-assisted methodology employing food models. In the fourth and final pass, the interviewer reviewed the recalled food intakes and confirmed these were accurate. Data were collected across all days of the week, with repeated recalls collected from a sub-sample of the participants (n = 55) at least two days after the initial recall to adjust for intra-subject variation when estimating the percentage at risk of inadequate nutrient intakes. To improve estimations of food quantities consumed, the researchers conducted orientation sessions with participants two days before the recall and provided participants with plates and bowls to eat from on the following day.

### 2.3. Pre-Processing and Cleaning of Data

#### 2.3.1. Food Consumption Data from 24HR Dietary Recall

We processed the 24HR recall raw data in Microsoft Excel. All food portion sizes were converted to grams. We calculated the amount of each ingredient consumed for mixed dishes/recipes based on preparation of standard recipes generated at the community level.

The data on food consumption quantities was not normally distributed with a right skew, and total daily consumption of individual food items revealed several quantities that were implausibly large. The food consumption data were then adjusted after being checked for data entry errors and outliers using the percentile approach. While no ideal methods exist to manage outliers in epidemiological datasets [22] the 95th percentile has been used to identify and treat outliers. In this study, food consumption quantities above the 95th percentile were reassigned the 95th percentile value, whereas the data for the lower outliers were not adjusted because, in the context of the study setting, they could reflect low food intake on that day.

#### 2.3.2. Household Apparent Food Consumption Data from IHS5

We converted all food consumption quantities recorded in standard (e.g., grams, millilitres) and non-standard units (e.g., pail, basin, heap) to the metric unit kilogram. The IHS5 food conversion factors from World Bank data, specific to Malawi, were used, and regional factors were applied according to the household’s location in Malawi [12]. Where relevant, we subtracted the non-edible portions of foods from the total quantity apparently consumed. For each food item, we divided the total quantity of food by seven to produce estimates of daily apparent household consumption.

The apparent food intakes from HCES were right-skewed, and visual inspection revealed multiple cases of implausibly high quantities of consumption for some food items. We adopted the approach of Tang et al. [10], whereby consumption quantities were log-transformed to approximate a normal distribution. Values exceeding five standard deviations above the mean were considered outliers and were replaced with the 95th percentile value—consistent with the method used to handle outliers in the 24HR recall dataset.

### 2.4. Food Composition Data

Nutrient conversion tables were constructed for the HCES and 24HR datasets through identifying ‘best fit’ matches with food items in the following food composition tables (FCTs): Malawi (69.2%) [23], Kenya (16.5%) [24], West Africa (12.8%) [25], and U.S. Department of Agriculture (USDA) (1.5%) [26]. The nutrient conversion tables did not include fortification values.

Food composition data were harmonized between the HCES and 24HR nutrient conversion tables, such that the same composition data were used for identical or similar items. Consistent FCT data were used to ensure that any variation in estimated nutrient intakes was not an artifact of variation between composition data for the same food items. However, due to differences in survey methods, the nutrient conversion tables differed in several respects, e.g., there was a larger number of food items reported in the 24HR, so that nutrient conversion table contains more data. We further adjusted the 24HR nutrient conversion table for cooking losses using USDA retention factors since the cooking methods were recorded.

### 2.5. Estimating Nutrient Intakes

We calculated energy and nutrient intakes from 24HR by multiplying the weight of each food item consumed by the matched nutrient contents per gram in the nutrient conversion table and summing across the whole diet for each participant. For the 24HR data, we adjusted the nutrient intake distributions for intra-subject variation. The nutrient intakes in the 24-h dietary recall were adjusted for usual intake using repeated recalls in Intake Modelling, Assessment, and Planning Program (IMAPP) software [27]. However, a single-day recall was used to generate median intakes for vitamin B12, as many entries were zeros and could not be processed in IMAPP. The low values for many individuals reflected no or limited consumption of vitamin B12-rich food sources, particularly animal-source foods.

Similarly, for each household in the IHS5, we calculated apparent energy and nutrient intakes by multiplying the individualized weight of each food item consumed by the matched nutrient content per gram in the nutrient conversion table, summing across the whole diet. Transformation for usual intake was not done for HCES apparent intakes (unlike 24HR), as we assumed that the 7-day intake adequately represented usual intakes [28].

### 2.6. Calculation of Households or Individuals at Risk of Inadequate Intakes

The estimated (apparent) nutrient intakes for both the HCES and 24HR were compared to the harmonized average requirements (HAR) [29] values for a nonpregnant, nonlactating adult woman aged 18–29 years to calculate the prevalence at risk of inadequate (apparent) intakes for vitamins A, B1, B2, B3, B6, B9, and B12, and zinc (Zn). We assumed a low bioavailability of Zn because diets are typically high in unrefined grains. For iron (Fe), we used the full probability approach to estimate the percentage at risk of inadequate intakes, assuming 5% iron bioavailability [30].

### 2.7. Fortification Scenarios

The study was designed, in part, to assess the ability to utilize HCES data to draw robust insights on the potential contribution of large-scale food fortification (LSFF) to micronutrient adequacy in Malawi, whereby current policy mandates the fortification of cooking oil and sugar with vitamin A and wheat flour with vitamins A, B1, B2, B3, B6, B9, and B12, Fe, and Zn [10]. As such, the analysis focused on these fortified nutrients to enable the modeling of fortification scenarios. We developed two fortification scenarios based on these three fortifiable products (oil, sugar, and wheat flour, including wheat flour products): assuming (a) no fortification and (b) an LSFF program with full compliance. We modelled the LSFF programme by changing the food composition values for the fortifiable products using assumptions specified by Tang et al. [10] under the improved fortification scenario, whereby it was assumed that oil, sugar, and wheat flour were fortified to legislated levels at the point of fortification, with a degree of degradation between production and household purchase. These fortification scenarios were applied to each dataset based on reported consumption of fortifiable foods. Specifically, for the HCES dataset, fortification was modelled based on reported household consumption of oil, sugar, and wheat flour. For the 24HR dataset, fortification was modeled based on reported individual consumption of these same products.

To compare vitamin A intake estimates derived from HCES and 24HR data under different fortification scenarios, we analyzed the distribution of intake values for each method and scenario. Intake distributions were visualized using density plots (also known as violin plots) to illustrate central tendency, spread, and skewness. For each fortification scenario, we compared the median intake between HCES and 24HR to assess systematic differences in intake estimation.

Given the non-normal distribution of intake data, we used the Mann–Whitney U test to statistically compare the distributions and intakes of fortifiable products between the two datasets. In addition, Pearson’s Chi-squared test was used to compare the proportion of households or individuals reporting consumption of fortifiable foods between the two datasets.

### 2.8. Creating a Comparable Subsample of the HCES

Dietary patterns vary spatially and by season in Malawi [10,31]. Thus, to increase the comparability between HCES and 24HR recall samples, we further subsampled the IHS5 data to match the season of the AHHA baseline survey, in June–July. Furthermore, we selected households with a WRA in residence. However, only 55 households met these eligibility criteria, which is small considering the random measurement error inherent in measuring dietary intakes using HCES methods [28]. We therefore examined month-to-month variation in apparent intakes of energy and the nine micronutrients under study as well as the month-to-month variation in apparent consumption of three fortifiable products (oil, sugar, and wheat flour) in grams per day per AFE. In broad terms, compared to June and July, estimated apparent intakes were similar in the months of March, May, August, and September, and we therefore widened the eligibility criteria to households responding in March-September inclusive. This resulted in a final sample size of 183 households. All data manipulation and calculation for HCES were performed using R (version 4.0.5, R Foundation for Statistical Computing, Vienna, Austria).

## 3. Results

### 3.1. HCES and 24HR Study Populations

Table 1 shows the social and demographic characteristics of the study populations. These comprise 183 households with nonpregnant women (18–49 years) from the HCES and 177 nonpregnant women (20–45 years) from the 24HR survey. The mean household size for HCES was smaller (n = 4.9) than for 24HR (n = 6.6), and the WRAs residing in households in the HCES sample were, on average, younger than participants in the 24HR. This was likely due to the recruitment criteria for the AHHA trial, which required the presence of a child aged 5–10 years in residence in the household.

### 3.2. Comparison of Dietary Intake Estimates

Median estimated intakes of energy and all micronutrients except vitamin B12 were higher in the 24HR survey than apparent intakes in the HCES. However, only energy and vitamin B3 (niacin) did not have overlapping interquartile ranges (IQR) (Table 2). Interquartile ranges were generally smaller for the 24HR usual intake than the HCES apparent intake; in the case of vitamin A, the 75th percentile (561.5 µg [micrograms] retinol activity equivalent [RAE]/day per AFE) was higher than that from the 24HR usual intake (462 µg RAE/day per person), despite the median value for 24HR being higher than for the HCES. The median value of vitamin B3 intake was nearly threefold higher in the 24HR survey than the median of apparent intake in the HCES. The median intake of vitamin B12 was zero in the 24HR survey, reflecting most participants who did not report any consumption of vitamin B12-containing foods (i.e., animal sources). Also, the interquartile range of vitamin B12 apparent intake was smaller in the HCES compared to intake in the 24HR survey.

### 3.3. Intake and Percentage of Households/Individuals Reporting Consumption of Fortified Foods

Table 3 shows the median daily consumption of sugar, cooking oil, and wheat flour (including wheat flour products) and the percentage of households/individuals consuming these items in the HCES and 24HR survey. The apparent consumption was lower in the HCES for all three food items compared with intakes in the 24HR survey (*p* < 0.001). However, the percentage of households/individuals reporting consumption of fortifiable foods was higher in the HCES for all three food items compared to the 24HR survey. The percentage of households/individuals reporting consumption of fortifiable foods, for both surveys, was highest for cooking oil (45% 24HR; 84% HCES), followed by sugar (28% 24HR; 43% HCES), and wheat flour and its products (12% 24HR; 32% HCES).

### 3.4. Large-Scale Food Fortification Contribution

Table 4 shows the estimated prevalence of apparent inadequate micronutrient intakes calculated using the HCES and inadequate micronutrient intakes in the 24HR survey. We calculated these for both the no fortification and fortification scenarios. Under the ‘no fortification’ scenario, the prevalence of micronutrient inadequacy fell within 20 percentage points between HCES and 24HR for Fe, Zn, vitamins B2, B9, and B12. Iron, Zn, and vitamin B2 all had a high prevalence of inadequacy (>75%) regardless of the method and fortification scenario. Vitamins B1 and B3 had a substantially lower prevalence of inadequacy (≤5%) in the 24HR survey compared to the HCES (33% to 74%).

Under the fortification scenario, the prevalence of inadequate (apparent) intakes was within 20 percentage points for the five micronutrients (i.e., Fe, Zn, vitamin A, vitamin B2, and vitamin B9) (Table 4). This set of micronutrients is like that in the no fortification scenario, except that vitamin A replaced vitamin B12, which was included in the no fortification scenario. Discordance in prevalence estimates was greatest for vitamin B3, followed by vitamin B6 and vitamin B1. Vitamin A showed the largest reduction in prevalence of inadequate (apparent) intakes with fortification, with a particularly large decline in the 24HR survey. The effects of fortification on prevalence estimates were typically small for other micronutrients, although a >10 percentage point reduction in prevalence was observed in the 24HR survey for vitamins B9 and B12.

## 4. Discussion

The purpose of this study was to determine whether estimates of micronutrient intake and prevalence of inadequacy derived from a national HCES were broadly comparable with those derived from a multi-pass individual-level 24-h dietary recall in rural Malawi. We also assessed the extent to which HCES provides a reliable source of dietary data to inform national LSFF policies, based on the comparability between estimates of dietary nutrient intake and risk of inadequate intake and the percentage of households/individuals reporting consumption of fortifiable food vehicles.

### 4.1. Discrepancies in Food and Energy Intakes Between 24HR and HCES Data

The median of apparent energy intakes was lower in the HCES than the 24HR. These findings are consistent with previous studies comparing HCES and 24HR data [28,32], and suggest that food consumption data in HCES under-represent dietary intakes of adult women. Our supposition that the low-reported energy intakes in the HCES result from underreporting of food consumption over seven days (rather than chronically energy-deficient diets) is supported by the observation in the 2015/16 Malawi Demographic and Health Survey that women in our study area typically have normal body mass indexes [14]. Therefore, we would expect energy intakes close to requirements, e.g., 2408 kcal/day for a WRA weighing 55 kg with a PAL of 1.85 or moderately active lifestyle [16]. The estimated consumption quantities of cereals, pulses, and nuts were all substantially lower in the HCES compared to the 24HR (Supplementary Figure S3A). The under-representation of food and energy consumption in HCES may be due to a range of factors, including challenges recalling food consumption for all household members in aggregate over a 7-day period and foods consumed away from home not being captured. These limitations of HCES are well documented in other contexts [33,34].

### 4.2. Prevalence of Dietary Micronutrient Inadequacies Using 24HR and HCES Data

In general, apparent micronutrient intakes in the HCES were lower than intakes in the 24HR, and we observed large discrepancies in prevalence estimates for the B vitamins. Vitamin B2 was an exception, where the prevalence of inadequacy was almost 100% using both HCES and 24HR across both fortification and no fortification scenarios. This is likely due to poor consumption of vitamin B2 rich foods such as meat and milk and milk products in Malawian households, which was reported in both datasets. For Fe and Zn, the similarity between the two datasets is most likely due to Malawian households’ overreliance on cereals, particularly maize, as a significant source of Fe and Zn [35]. Otherwise, the prevalence of inadequacy estimated from HCES was consistently higher than that estimated from 24HR, and in the case of vitamins B1, B3, and B6, by more than tenfold. For vitamin B3, the larger difference between 24HR and HCES estimates likely stemmed from the HCES food list description of certain food items such as fish, pulses, and nuts, which were insufficiently detailed to support robust matches to foods listed in the FCT (Supplementary Figures S2 and S3A–I). For example, fish in the Malawi HCES are described as fresh or dry with no other descriptions to guide FCT matching. Vitamins B1 and B6 provide a similar case. The discrepancies were large enough that different inferences regarding population nutritional status would be drawn from HCES data versus 24HR data, highlighting that caution is required when relying on a single source of information on population-level risk of inadequate micronutrient intake.

The median intake of vitamin B12 estimated from 24HR (although not adjusted for usual intake) was lower than that estimated from the HCES, yet we observed a lower prevalence of inadequacy in the 24HR than in the HCES (Table 4, no fortification), although both estimates exceeded 70%. This was due to the episodic consumption of vitamin B12-rich foods such as meat and meat products, which are more likely to be captured in a 7-day recall of a whole household’s diet rather than a single 24HR of an individual. Although animal-source food consumption was more frequently reported in HCES than 24HR, the individualised quantities were small such that for the majority of households, vitamin B12 apparent intakes were still inadequate. Therefore, these findings suggest that HCES-derived apparent intake estimates for Fe, Zn, vitamins A, B9, and B2 were reasonably robust, and widespread prevalence of inadequate intakes would be inferred from both datasets.

### 4.3. Using 24HR and HCES Modelling Outputs to Inform LSFF Decision-Making

The potential contribution of LSFF for reducing the risk of inadequate intake was substantially greater for vitamin A than other micronutrients, and this was consistently identified using HCES and 24HR data. This is due to the limited consumption of wheat flour and wheat-containing products, while cooking oil and sugar (which are fortified with vitamin A but not other micronutrients) were widely consumed. Between 2000 and 2009, Malawi ranked 41st among 48 Sub-Saharan African countries in terms of per capita wheat consumption (6.48 kg/year) compared to a regional average of 21.2 kg/year [36]. The most widely consumed fortifiable product was cooking oil, followed by sugar, and this observation was consistent in both HCES and 24HR despite differences in coverage and intake quantities. Together, the two commodities contributed a high percentage of the estimated intake of vitamin A (Supplementary Figure S1). Fortification of wheat flour and products led to a negligible decrease in estimated prevalence at risk of inadequate vitamin A intakes, consistent with findings reported by Tang et al. (2022) [10]. This was due to low coverage and intake of wheat flour and its products in both datasets. Maize fortification was not modelled because maize consumed in Malawi is mostly processed in community-based maize mills where LSFF is not implemented [37].

The implications from this analysis are that similar programmatic decisions on the choice of food fortification vehicles will be made based on either the HCES or 24HR data. Although HCES likely under-represents food item and nutrient intakes, it may provide a better measure of fortification vehicle and program coverage due to the longer recall period. The 7-day recall period in HCES would likely pick up more households consuming the fortifiable foods, including episodic foods, as opposed to the 24HR methodology, which is not recommended for estimating coverage. Thus, analysis of HCES and 24HR surveys may provide complementary insights for informing LSFF programmatic decisions. Additional data on population biomarker status and fortificant concentrations in food vehicles sampled at markets or homes would also support policy and program decisions. The biomarker data would be accompanied by programmatic evidence on access and affordability of fortified foods and geographic and socioeconomic disparities to ensure that LSFF initiatives reach those most at risk of micronutrient deficiencies [38].

### 4.4. Limitations of This Study

While we attempted to match the two populations as much as possible, our study used two unrelated samples where family size was different, and we were unable to account for differences in socio-economic profiles, as data were insufficient to allow for classification by wealth quintiles. This may have introduced selection bias; for instance, if one sample disproportionately included wealthier households, influencing food availability and dietary diversity. In addition, we worked with secondary data for this study, thus inheriting limitations from the parent datasets and methodologies associated with each survey. HCES may provide a proxy measure of food consumption and provide useful dietary insights, including estimating and assessing the potential role of LSFF. However, there are important limitations to this approach. First, food consumed away from home was not captured, which may have led to an underestimation of total intake. Second, individual-level estimates were derived from household-level data using the AFE approach, which involves crude assumptions about intra-household food distribution. While this approach provides a standardized reference, it may not reflect the actual needs of all household members or WRA. Finally, anthropometric and physical activity data were not collected for various demographic groups in HCES. This limited our ability to estimate individual energy requirements accurately and to contextualize reported energy intakes, leading to misclassification bias, as we had to rely on assumed average body weights and PALs.

Nevertheless, we harmonized the two datasets to make them comparable by residence (Kasungu rural), age (women of reproductive age), and season (post-harvest months). Our data were also comparable based on lactating status and the education level of the household head, which are known to influence dietary intake. We further harmonized the food composition tables for all identical food items in the two datasets. This step was critical to ensure that any variation we observed between the apparent intake and intake of micronutrients was not an artifact of variation between the two FCTs but the data collection tools (HCES and 24HR). The 24HR dataset included repeat recalls for a sub-sample of the women, which allowed for adjustment of day-to-day variability to estimate usual intakes. Without this adjustment, estimates would have been inflated due to day-to-day variability. The use of the HCES with data for 7-days allowed for approximate usual intake estimates, and considering our population was rural, the proportion of meals eaten away from home was likely very low. Even if they were captured, there are serious concerns because the current survey modules for estimating food consumed away from home underestimate energy and macronutrient intakes in low-and middle-income countries [39]. Where food eaten away from home has been reported, there is a lack of standardized definitions, making comparability across studies difficult [40]. Together, these methodological steps helped reduce potential sources of bias and allowed for a more meaningful comparison of nutrient intake and inadequacy estimates between the two data sources.

## 5. Conclusions

In Malawi, HCES offers a potentially practical and scalable tool for estimating the adequacy of dietary nutrient intake for rural WRA (e.g., Fe, Zn, vitamins B2 and B9), types and frequency of consumption of fortified foods, and potential programmatic entry points. However, our findings indicate that HCES may systematically underestimate absolute nutrient intakes compared to 24HR data. To enhance the utility of HCES for nutrition applications, improvements to survey design are needed. These include more precise descriptions of food items (e.g., fish) and capture of foods eaten away from home. Modifications to the survey design could be pilot tested prior to adoption and compared against individual-level 24HR to assess the value for reducing measurement error. In addition, collecting anthropometric and physical activity data would allow for more precise estimation of energy requirements and better contextualization of intake data. Together, these approaches can help ensure that both HCES and 24HR data are used effectively and complementarily in guiding LSFF initiatives. 

## Figures and Tables

**Table 1:** Social and demographic characteristics of the study populations for the HCES and 24HR data from Kasungu district Malawi.

Variable	HCES (n = 183)	24HR (n = 177)
Household size, mean (SD)	4.9 (1.8)	6.6 (1.9)
Highest education level of household head (%)	What is the highest educational qualification you have acquired?	What is the highest level of education completed by the adult woman participant?
Primary/none	74.9	82.2
Secondary	18.6	15.6
More than secondary	-	2.2
Don’t know	6.6	-
Age groups in years (%)	n = 207 (all adult women in sample households)	
18–19	16.4	-
20–29	41.5	32.2
30–39	26.1	43.3
40 and more	15.9	24.4
Lactating women (%)	Proportion of households with child <24 months	Proportion of women reporting lactating
	27.3	33.3
Age groups for breastfeeding children in months (%)	Proportion of households with child in age range	Proportion of women with child in age range
0–6	24.0	18.6
7–11	18.0	25.4
12–23	58.0	49.2
≥24	-	6.8
Age of breastfeeding children, mean (SD)	Age of children <24 months in the household	Age of children (in months) of lactating women
	12.2 (6.8)	13.0 (6.6)

**Table 2:** Comparison of median (IQR) apparent intakes of energy and micronutrients among households in Kasungu district in the HCES dataset and daily intake of energy and micronutrients among WRA from a 24HR dataset.

Variable	HCES	24HR ^1^	HAR—WRA ^2^
Energy, kcal	1603 (1145, 2063)	2537 (2211, 2903)	-
Iron, mg	13.2 (8.8, 20.8)	17.0 (14.3, 20.1)	22.4
Zinc, mg	5.9 (3.9, 9.1)	9.1 (8.0, 10.4)	10.2
Vitamin A, μg RAE	263.7 (111.4, 561.5)	411.8 (367, 462)	490
Vitamin B1, mg	1.2 (0.8, 2.0)	1.6 (1.4, 1.9)	0.9
Vitamin B2, mg	0.5 (0.3, 0.6)	0.7 (0.6, 0.7)	1.3
Vitamin B3, mg	7.8 (5.4, 11.1)	20.0 (16.6, 23.9)	11
Vitamin B6, mg	1.2 (0.8, 1.8)	1.7 (1.6, 1.8)	1.3
Vitamin B9, μg Dietary Folate Equivalent	246.4 (181.3, 407.4)	304.6 (264, 349)	250
Vitamin B12, µg	0.4 (0.1, 1.1)	0.0 (0.0, 1.4)	2

^1^ Median (IQR) values for all micronutrients except for vitamin B12 were adjusted for usual intake using IMAPP. **Note:** Values from the HCES are individualized and expressed per day per adult female equivalent; Results are under a ‘no fortification’ scenario. ^2^ Harmonized Average Requirement (HAR) values were sourced from Allen et al. [29]. For iron, requirements were based on low dietary iron bioavailability. For zinc, values reflect requirements assuming an unrefined diet.

**Table 3:** Comparison of median (IQR) of (apparent) intake consumption quantity and percentage of households/individuals reporting consumption of fortifiable foods (coverage) between HCES and 24HR.

Food Vehicle	HCES (n = 183)	24HR (n = 177)	*p*-Value ^1^
Sugar			
Intake (g/day)	17.9 (5.6, 29.3)	26.0 (20.1, 36.1)	<0.001
Coverage (%)	42.6	27.7	0.003
Oil			
Intake (g/day)	5.7 (2.6, 10.7)	21.2 (10.0, 37.7)	<0.001
Coverage (%)	83.6	45.2	<0.001
Wheat flour and products			
Intake (g/day)	12.6 (6.8, 18.0)	57.0 (44.8, 77.1)	<0.001
Coverage (%)	32.2	11.9	<0.001

^1^ Comparison (intake) done using Mann–Whitney U test; Comparison (%) done using Pearson’s Chi-squared test.

**Table 4:** Comparison of percentage of households with apparent inadequate intakes of micronutrients in the HCES dataset and percentage of women at risk of inadequate micronutrient intakes from the 24HR dataset in Kasungu district under two scenarios of LSFF.

	No Fortification			Fortification		
Nutrient	24HR (%)	HCES Apparent Intake (%)	% Pt. Difference	24HR (%)	HCES Apparent Intake (%)	% Pt. Difference
Iron	90.1	85.4	4.7 *	90.0	85.3	4.7 *
Zinc	83.3	80.3	3.0 *	82.7	79.2	3.5 *
Vitamin A	95.5	71.0	24.5	44.8	48.6	3.8 *
Vitamin B1	2.5	33.9	−31.4	1.7	33.9	−32.2
Vitamin B2	100	98.9	1.1 *	100	98.9	1.1 *
Vitamin B3	5.3	73.8	−68.5	4.7	73.2	−68.5
Vitamin B6	19.7	55.2	−35.5	19.0	54.6	−35.6
Vitamin B9	49.3	52.5	−3.2 *	37.1	48.6	−11.5 *
Vitamin B12	70.4	88.0	−17.6 *	60.2	88.0	−27.8

Note: % pt. = percentage point. Values within a 20 percentage point difference are highlighted with asterisks (*).

## Data Availability

The household consumption and expenditure survey (HCES) data were collected as part of Malawi’s IHS5 and are available from the World Bank repository at https://microdata.worldbank.org/index.php/catalog/3818 (accessed on 14 June 2023). The dietary data from the AHHA trial will be made available via the LSHTM Data Compass repository at https://doi.org/10.17037/DATA.00003400.

## Supplementary Materials

The following supporting information can be downloaded at: https://www.mdpi.com/article/10.3390/nu17152485/s1, Figure S1: Distribution of data, and comparison of median vitamin A intake estimated using HCES and 24HR by each fortification vehicle (A) no fortification, (B) fortification using wheat flour, (C) fortification using sugar and (D) fortification using cooking oil. Figure S2: Average daily food group contribution to vitamin B3 for the 24HR (mg/day per person) and the HCES (mg/day per AFE). Figure S3A–I: Average food group contribution to energy and micronutrient consumption, per person per day for the 24HR and per AFE per day for the HCES.
