# Supplementary material for: Use of Household Apparent Food Intake Data to Estimate Micronutrient Inadequacy in Comparison to the 24-h Recall Data Among Women of Reproductive Age in Kasungu District, Malawi
Source: Nutrients. 2025 Jul 30;17(15):2485. doi: 10.3390/nu17152485 (PMC12348564; doi:10.3390/nu17152485)
Supplement: Supplementary file 1 [file nutrients-17-02485-s001.zip › Supplementary_Figure S1 (1).pdf]

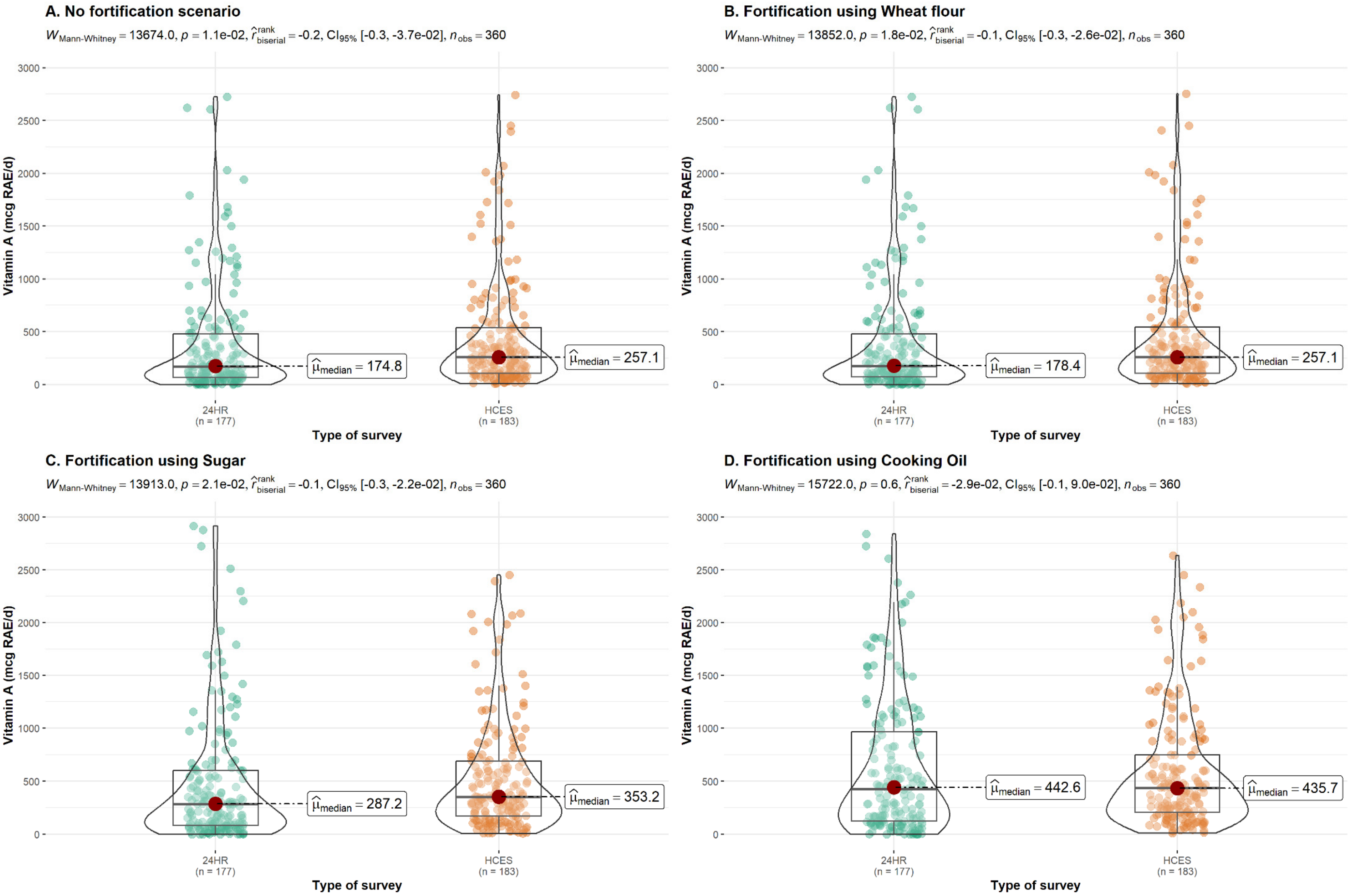

Supplementary Figure S1. Distribution of data, and comparison of median vitamin A intake estimated using HCES and 24HR by each fortification vehicle (A) no fortification, (B) fortification using wheat flour, (C) fortification using sugar and (D) fortification using cooking oil.
