# Supplementary material for: Use of Household Apparent Food Intake Data to Estimate Micronutrient Inadequacy in Comparison to the 24-h Recall Data Among Women of Reproductive Age in Kasungu District, Malawi
Source: Nutrients. 2025 Jul 30;17(15):2485. doi: 10.3390/nu17152485 (PMC12348564; doi:10.3390/nu17152485)
Supplement: Supplementary file 1 [file nutrients-17-02485-s001.zip › Supplementary_Figure S2 (1).pdf]

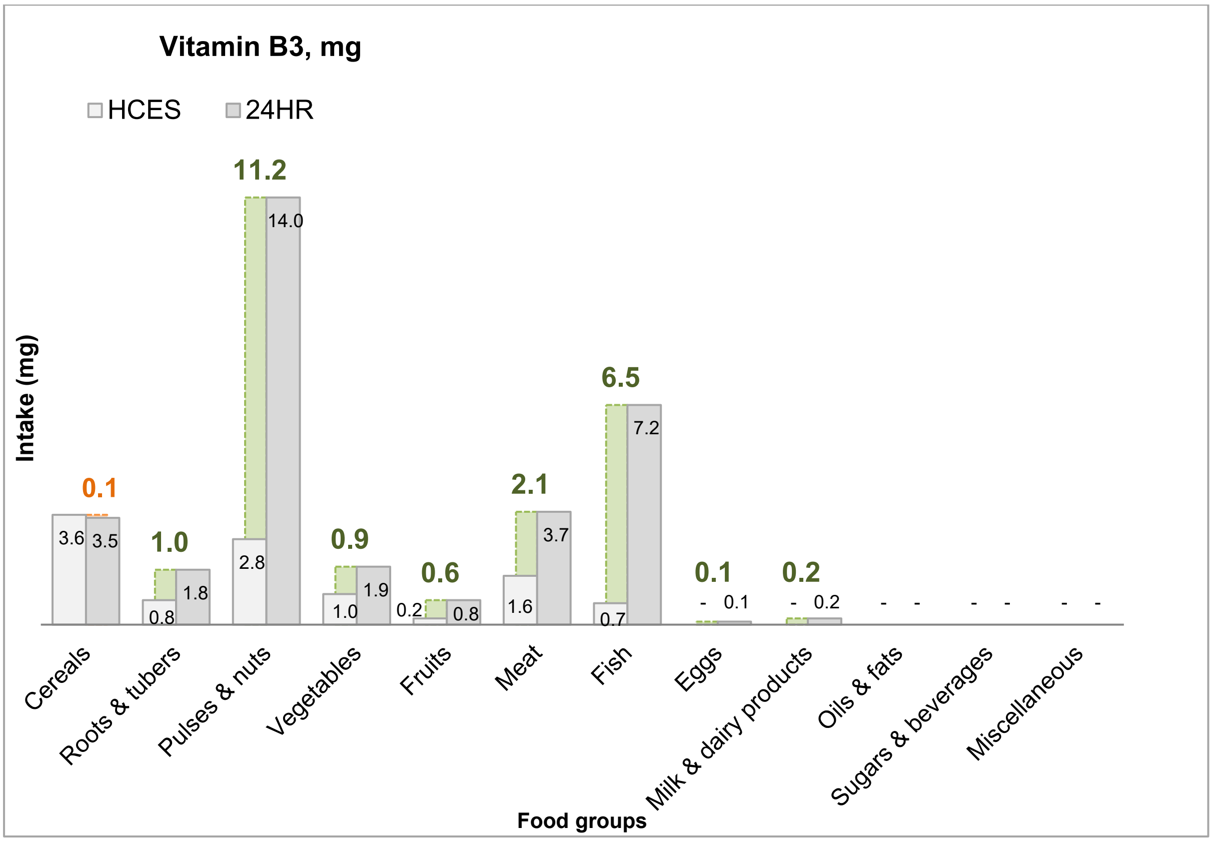

Supplementary Figure S1. Average daily food group contribution to **vitamin B3** for the 24HR (mg/person/day) and the HCES (mg/AFE per day)

Key: Green= 24HR intakes greater than HCES; Orange= HCES intakes greater than 24HR
