# Supplementary material for: Use of Household Apparent Food Intake Data to Estimate Micronutrient Inadequacy in Comparison to the 24-h Recall Data Among Women of Reproductive Age in Kasungu District, Malawi
Source: Nutrients. 2025 Jul 30;17(15):2485. doi: 10.3390/nu17152485 (PMC12348564; doi:10.3390/nu17152485)
Supplement: Supplementary file 1 [file nutrients-17-02485-s001.zip › Supplementary_Figure S3 (A_I).pdf]

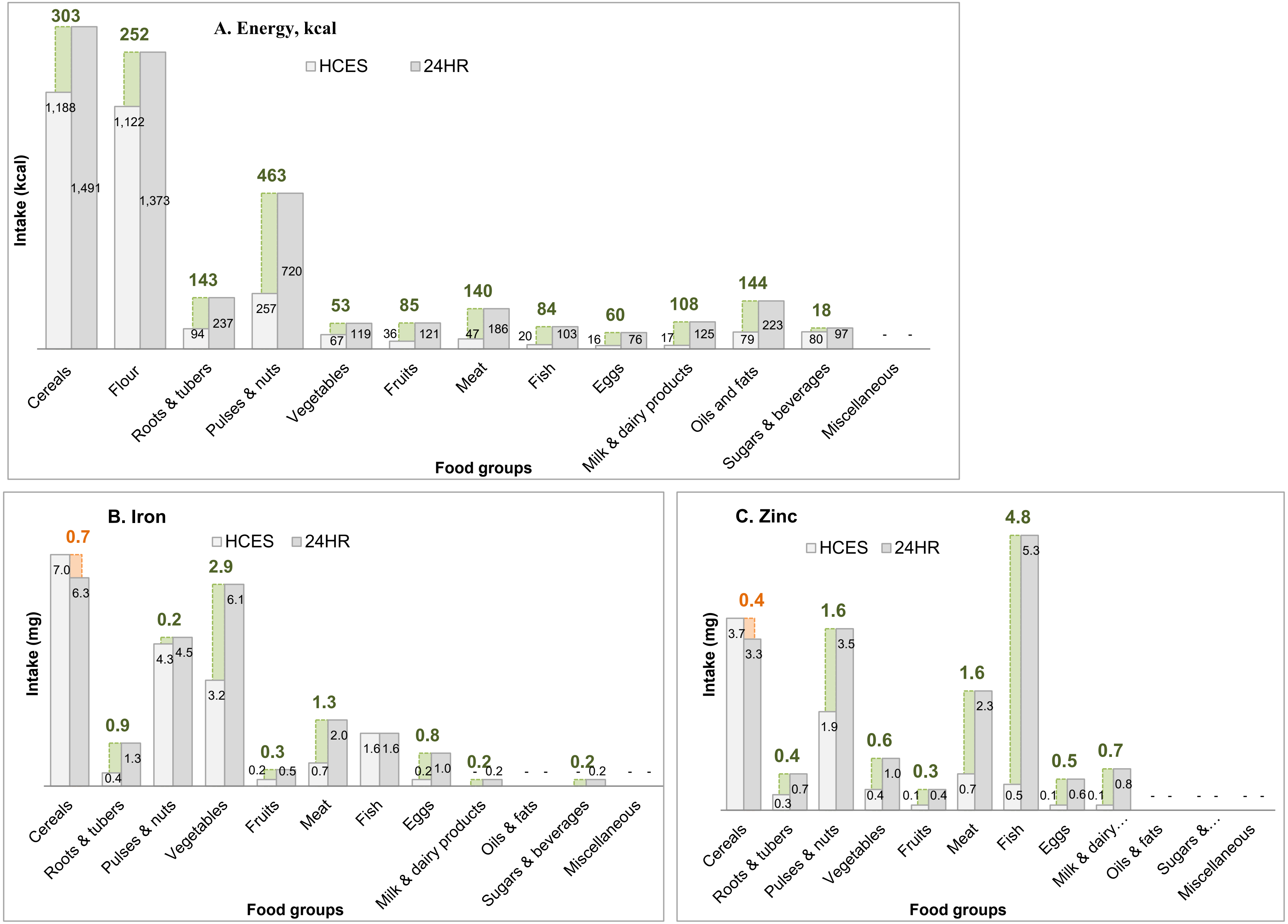
A. Energy, kcal
HCES
24HR
Intake (kcal)
Food groups
Cereals
Flour
Roots & tubers
Pulses & nuts
Vegetables
Fruits
Meat
Fish
Eggs
Milk & dairy products
Oils and fats
Sugars & beverages
Miscellaneous
303
1,188
1,491
252
1,122
1,373
143
94
237
463
257
720
53
67
119
85
36
121
140
47
186
84
20
103
60
16
76
108
17
125
144
79
223
18
80
97
B. Iron
HCES
24HR
Intake (mg)
Food groups
0.7
7.0
6.3
0.9
0.4
1.3
0.2
4.3
4.5
2.9
3.2
6.1
0.3
0.2
0.5
1.3
0.7
2.0
1.6
1.6
0.8
0.2
1.0
0.2
0.2
0.2
0.2
Cereals
Roots & tubers
Pulses & nuts
Vegetables
Fruits
Meat
Fish
Eggs
Milk & dairy products
Oils & fats
Sugars & beverages
Miscellaneous
C. Zinc
HCES
24HR
Intake (mg)
Food groups
0.4
3.7
3.3
0.4
0.3
0.7
1.6
1.9
3.5
0.6
0.4
1.0
0.3
0.1
0.4
1.6
0.7
2.3
4.8
0.5
5.3
0.5
0.1
0.6
0.7
0.1
0.8
Cereals
Roots & tubers
Pulses & nuts
Vegetables
Fruits
Meat
Fish
Eggs
Milk & dairy…
Oils & fats
Sugars &…
Miscellaneous

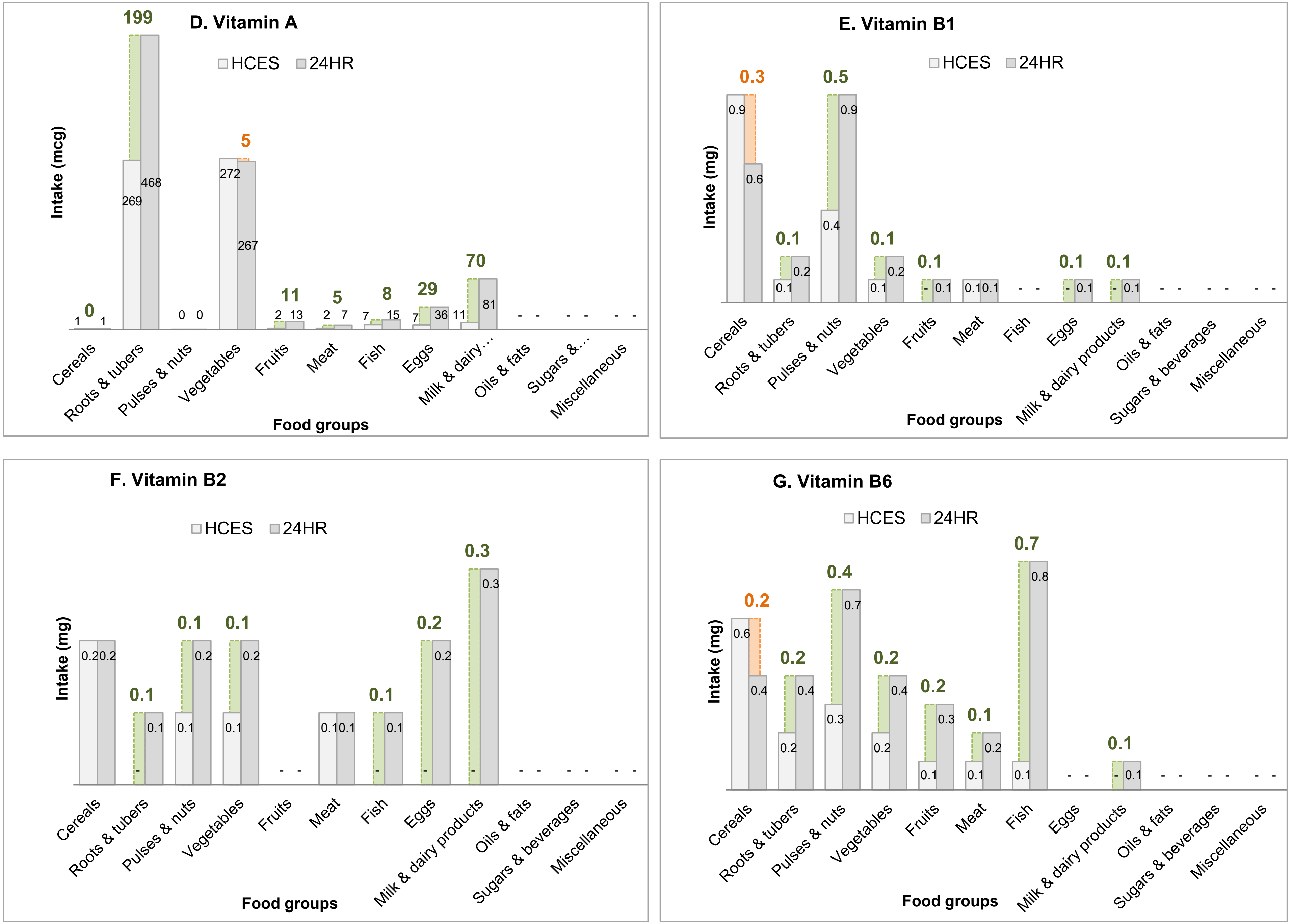

D. Vitamin A
HCES
24HR
Intake (mcg)
Food groups
Cereals
Roots & tubers
Pulses & nuts
Vegetables
Fruits
Meat
Fish
Eggs
Milk & dairy…
Oils & fats
Sugars &…
Miscellaneous
0
1
1
199
269
468
0
0
5
272
267
11
2
13
5
2
7
8
7
15
29
7
36
70
11
81
E. Vitamin B1
HCES
24HR
Intake (mg)
Food groups
Cereals
Roots & tubers
Pulses & nuts
Vegetables
Fruits
Meat
Fish
Eggs
Milk & dairy products
Oils & fats
Sugars & beverages
Miscellaneous
0.3
0.9
0.6
0.1
0.1
0.2
0.5
0.4
0.9
0.1
0.1
0.2
0.1
0.1
0.10.1
0.1
0.1
0.1
0.1
F. Vitamin B2
HCES
24HR
Intake (mg)
Food groups
Cereals
Roots & tubers
Pulses & nuts
Vegetables
Fruits
Meat
Fish
Eggs
Milk & dairy products
Oils & fats
Sugars & beverages
Miscellaneous
0.20.2
0.1
0.1
0.1
0.1
0.2
0.1
0.1
0.2
0.10.1
0.1
0.1
0.2
0.2
0.3
0.3
G. Vitamin B6
HCES
24HR
Intake (mg)
Food groups
Cereals
Roots & tubers
Pulses & nuts
Vegetables
Fruits
Meat
Fish
Eggs
Milk & dairy products
Oils & fats
Sugars & beverages
Miscellaneous
0.2
0.6
0.4
0.2
0.2
0.4
0.4
0.3
0.7
0.2
0.2
0.4
0.2
0.1
0.3
0.1
0.1
0.2
0.7
0.1
0.8
0.1
0.1

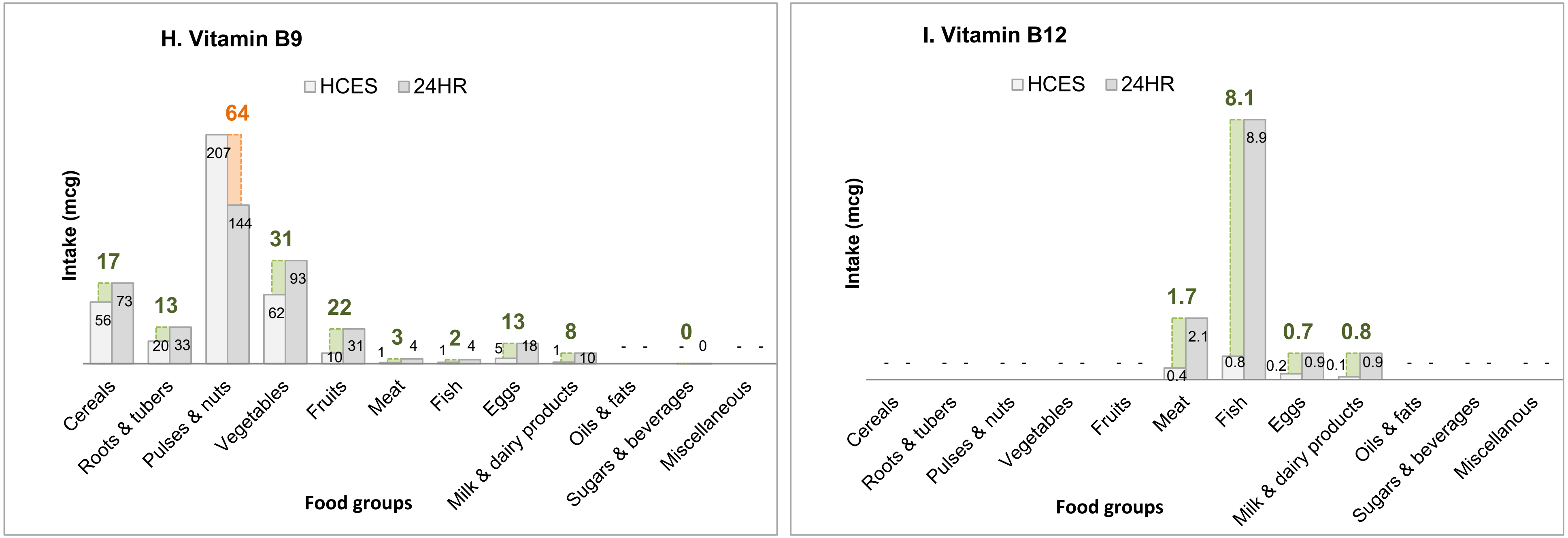

Supplementary Figure S1. (A-I) Average food group contribution to energy and micronutrient consumption, per person per day for the 24HR and per AFE per day for the HCES

Key: Green= 24HR intakes greater than HCES; Orange= HCES intakes greater than 24HR
